# Prenatal care utilization in pregnant women who consider but do not have abortions

**DOI:** 10.1186/s12884-021-04343-x

**Published:** 2022-01-21

**Authors:** Marika Toscano, Jillian Wood, Sara Spielman, Rita Ferri, Natalie Whaley, Neil S. Seligman

**Affiliations:** 1 Department of Obstetrics & Gynecology, University of Rochester Medical Center, 601 Elmwood Ave, Box 668, Rochester, NY 14642 USA; 2 Department of Public Health Sciences, University of Rochester Medical Center, 265 Crittenden Blvd, Rochester, NY 14642 USA; 3 School of Medicine and Dentistry, University of Rochester Medical Center, 601 Elmwood Ave, Rochester, NY 14642 USA

**Keywords:** Prenatal care utilization, Termination of pregnancy, Pregnancy ambivalence, Postpartum contraception

## Abstract

**Background:**

Over half of all pregnancies in the United States are unintended, and 18% result in termination of pregnancy (TOP). Some women seek TOP, but ultimately continue their pregnancy. Data are limited about their utilization of prenatal care and their perinatal outcomes. Our primary outcome was to investigate differences in guideline-based prenatal care utilization in women who consider but do not have an abortion.

**Methods:**

Retrospective cohort study of patients having obstetrical dating ultrasound (US) from 2011–2018 at a single academic medical center that offers TOP. Contemplators completed US with intention of TOP but instead continued the pregnancy to live birth. A 2:1 group of non-contemplators completed US and continued to live birth. A prenatal care utilization scoring system was used to compare groups. Secondary outcomes investigated differences in adverse pregnancy outcomes and postpartum care.

**Results:**

There were 94 contemplators and 183 non-contemplators. Inadequate prenatal care utilization initially was more common in contemplators than non-contemplators (62.8% vs 85.8%, *p* < 0.01) but was not significant after adjustment (aOR 1.0, 95% CI 0.40 – 2.56). There were no differences in adverse obstetric or neonatal outcomes. Contemplators were significantly more likely to have a postpartum contraceptive method (PPCM) upon hospital discharge (aOR 4.8, 95% CI 1.16 – 20.0) and significantly more likely to use a highly-effective PPCM (aOR 6.4, 95% CI 2.34 – 17.4).

**Conclusions:**

Reversal of intention for TOP is not associated with differences in prenatal care utilization, but is associated with increased uptake of postpartum contraceptive method.

**Supplementary Information:**

The online version contains supplementary material available at 10.1186/s12884-021-04343-x.

## Background

Excluding spontaneous abortions, 18.4% of pregnancies in the United States in 2017 ended in therapeutic abortion [1]. While abortions are common and legal in the US, there are still many barriers that pregnant people face in accessing abortion care, including lack of insurance coverage, not knowing how to obtain care, inability to afford or coordinate transportation, missed wages, misinformation or delays in access arising from crisis pregnancy centers, and policy-related barriers restricting access [2–5]. Between 7.2% and 11% of pregnant people considering termination ultimately continue their pregnancies [6, 7]. This number includes pregnant people facing policy-related financial or logistical barriers to accessing abortion care, 2% of pregnant people who are denied care due to having a gestational age beyond their clinic’s limit (turnaways) and 2–8% of pregnant people who decide not to get an abortion due to changes in their own personal decision-making [4, 6–8].

It cannot be assumed that women who initially consider or seek abortion services have unplanned pregnancies [9], though there are similarities and overlap between these populations. Pregnancy intendedness has been shown to predict pregnancy-related behaviors. Studies of people with unintended pregnancies report they are more likely to present at later gestational ages for prenatal care and are more likely to have fewer antenatal visits compared to those with planned pregnancies [10–14].

There is a small but expanding body of literature on the subpopulation of pregnant people who consider abortion, but ultimately continue with pregnancy. Most of these studies investigate the experiences of turnaways, including effects on mental health, educational attainment, poverty, neonatal outcomes, and subsequent pregnancy outcomes [7, 15–21]. However, there are few data published on prenatal care utilization in this subgroup of pregnant people during their ongoing pregnancy. Studies have reported initiation of prenatal care at later gestational ages [22], though some authors report that change in decision-making towards abortion is protective [23]. As compared to those who enter prenatal care without considering abortion, these people have considerably increased health and social service needs which can affect prenatal care utilization [24].

This retrospective observational study aims to fill a gap in research on prenatal care utilization by pregnant people who consider termination, but whose pregnancies end in live birth. A comparison of completeness of guideline-based prenatal care between abortion contemplators and non-contemplators was the primary outcome. Secondary measures including obstetric and neonatal outcomes, postpartum contraception usage and utilization of postpartum care were also compared.

## Methods

### Study design and setting

This is a retrospective cohort study. All patients received obstetric care at a single tertiary-care medical center in New York State that provides abortion services up to 24 weeks’ gestation of pregnancy for a large geographic catchment area. Data were collected from patients who had a live birth from January 1, 2011 to July 31, 2018.

The exposed group were patients contemplating a termination of pregnancy (TOP) but ultimately continuing on to a live birth due to reversal of decision for TOP for any reason, including late gestational age at presentation. The non-exposed group were patients during the same period who had not contemplated TOP and continued on to a live birth. The primary outcome of interest was difference in prenatal care utilization between groups. Secondary outcomes included differences in individual measures of prenatal care utilization, multiple adverse obstetrical and neonatal outcomes and postpartum contraception and care utilization between groups. The Strengthening the Reporting of Observational Studies in Epidemiology (STROBE) guidelines for reporting observational studies were used in study design and manuscript preparation [25]. The study was approved by the lead author’s Institutional Review Board at University of Rochester Medical Center.

### Patient selection

Eligible patients were identified by searching the local ultrasound database (AS-OBGYN, AS Software Inc, Englewood Cliffs, NJ, USA) for patients undergoing obstetric ultrasound for pregnancy confirmation and dating during relevant dates. “Contemplators” were identified using combined results of database search for billing codes for TOP and for sonographer descriptive comments (“TOP”, “abortion”) to capture the largest number of eligible subjects. “Non-contemplators” were identified as contemporary patients without these billing codes or descriptive comments. Patients were ineligible if they underwent termination of pregnancy, experienced first trimester pregnancy loss/ectopic pregnancy or previable stillbirth, or had incomplete prenatal care and delivery records available for review. Patients were included if they were age 13–55 with single or multiple gestations.

To decrease misclassification bias, further eligibility was then assessed through linkage of individual patient medical record number from the AS-OBGYN database to the local electronic medical record (Epic Systems Corporation, Verona, WI, USA) to confirm the ultrasound was performed in the setting of a desired termination (contemplators) or in the absence of desire for termination (non-contemplators) and that the same pregnancy was continued on to live birth.

Non-contemplators were matched to contemplators in approximately a 2:1 ratio. All eligible contemplators were included. Non-contemplators were selected as a convenience sample of patients obtaining a dating ultrasound during the same time frame.

A flow diagram demonstrating subject eligibility and selection is shown in additional Fig. 1.

### Variables of interest

Data for all variables were collected from the ultrasound database, electronic medical record and New York State Birth Certificate and Statewide Perinatal Data System (NYS-BC/SPDS). Accuracy of data abstraction was confirmed by random review by a senior study team member (M.T.).

#### Demographic information

Demographic information to characterize the two study groups was collected. The demographic variable “low educational attainment” was defined as achieving a high school diploma/GED or less. The demographic variable “prenatal depression” was assessed through self-reported response to question in NYS-BC/SPDS record asking “during pregnancy, would you say that you were: 1) not depressed at all, 2) a little depressed, 3) moderately depressed, 4) very depressed, 5) very depressed and had to get help” with answer choices 2–5 considered positive for presence of prenatal depression.

#### Prenatal Care Utilization  Score

**Fig. 1:**
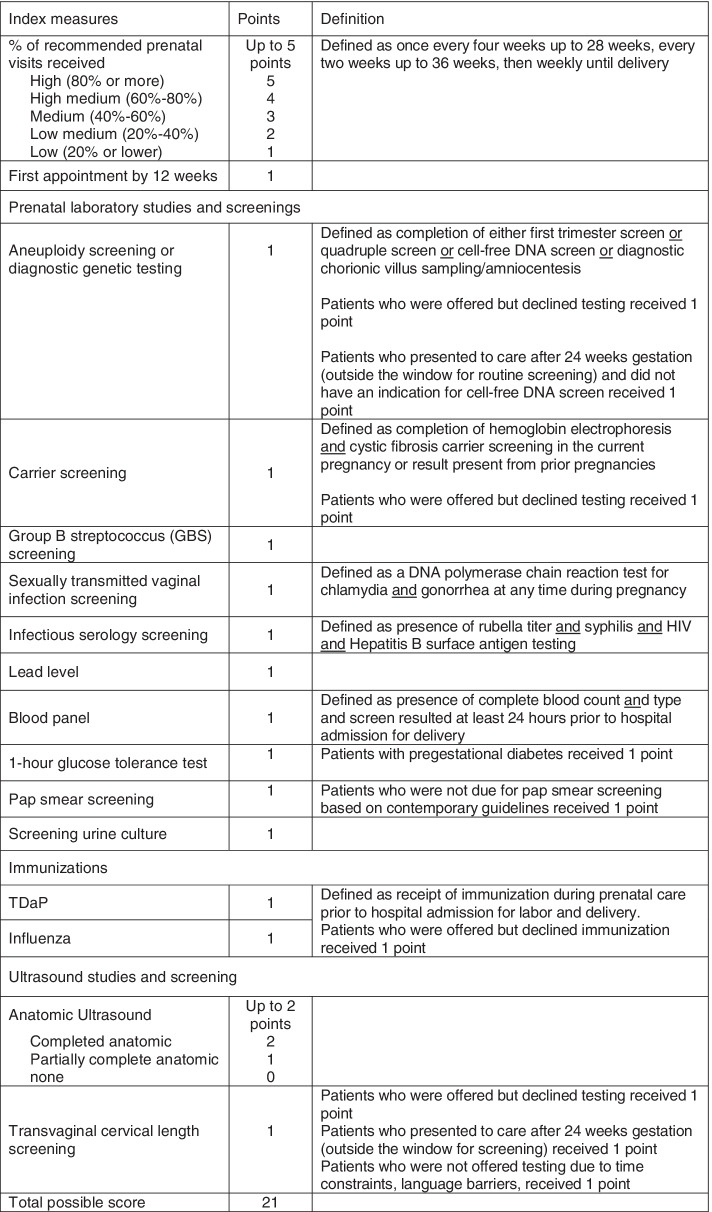
Overview of Scoring System Developed to Assess Guideline-Based Prenatal Care Utilization

Difference in guideline-based prenatal care utilization between groups was determined using a prenatal care utilization scoring system developed for this study. Variables included in the scoring system and their assigned point values are outlined in Fig. 1. Prenatal care was considered adequate when 16 or more points were achieved out of a total of 21 possible points, equating to ~ 75% completion.

Because of prior publications reporting that unintended pregnancy is correlated with late presentation to prenatal care and fewer antenatal visits, a sub-analysis was planned by removing two variables (“% of recommended prenatal visits received” and “first appointment by 12 weeks”) yielding a maximum score of 15. A score $\geq$ 11 points/15 points (~ 75% completion) was considered inadequate prenatal care.

#### Secondary outcomes

Secondary aims of this study were to compare individual measures of guideline-based prenatal care utilization, as well as obstetric and neonatal outcomes, between contemplator and non-contemplator groups.

Another secondary aim was also to compare postpartum contraception intention between contemplator and non-contemplator groups. Patients were classified as having a postpartum contraception intention if a specific contraceptive method was documented in the electronic medical record prior to discharge from the hospital admission related to the delivery. Type of immediate postpartum contraception utilized was compared between groups and was stratified into highly effective (long-acting reversible contraceptive placed or depo-provera administered prior to discharge, permanent sterilization, partner vasectomy) or all other methods.

Postpartum care utilization, including presentation to postpartum care visit and rates of postpartum depression screening (by Edinburgh Postnatal Depression Scale) and were also compared between groups.

#### Statistical analyses

Demographic variables were compared between contemplator and non-contemplator groups using Fisher’s exact tests and Chi-square tests. Distribution of continuous data was established using the Shapiro–Wilk test followed by Mann–Whitney U test for non-parametric data or Student’s t-test for parametric data. The primary outcome of interest, prenatal care utilization score, was compared between contemplators and non-contemplators using chi-square tests or Mann–Whitney-U tests. Multiple logistic regression analysis was performed to estimate the odds of having a total prenatal care utilization score ≥ 16 as a function of contemplators versus non-contemplators after adjusting for potential confounding variables. Results were calculated as odds ratios (ORs) with 95% confidence intervals (CIs). The data contained at least ten events for each variable entered into the logistic regression model [26]. Confounding variables were identified as maternal demographic variables with significant differences (*P* < 0.05) in the above bivariate analyses and included race, age, insurance type, education, prior termination, tobacco use in pregnancy, substance use in pregnancy, depression in pregnancy, STI in pregnancy, and gestational age at presentation to prenatal care. Similar analyses were performed to examine all secondary outcomes. All statistical analyses were performed using IBM SPSS Statistics (Version 27.0). Results were considered significant at two-sided *P* value < 0.05.

## Results

### Demographics

Data were collected on 94 contemplators and 183 non-contemplators, for a total of 277 participants. Statistically significant differences were noted between contemplators and non-contemplators in several maternal characteristics (see Table 1).

**Table 1:** Demographic Characteristics of the Study Population

Characteristics	Contemplators n (%)	Non-Contemplators n (%)	*P*-value^a^
Total, *n* = 277	*n* = 94	*n* = 183	
Race			< 0.01
White	28 (29.8%)	111 (60.7%)	
Black	62 (66.0%)	51 (27.9%)	
Other	4 (4.3%)	21 (11.5%)	
Hispanic ethnicity	8 (8.5%)	16 (8.7%)	0.95
Age^b^, (years)	24 (21–28)	29 (24–34)	< 0.01
Public insurance	80 (85.1%)	87 (47.5%)	< 0.01
Low educational attainment	69 (73.4%)	73 (39.9%)	< 0.01
Multiparous	62 (66%)	112 (61.2%)	0.44
Number of prior pregnancies^b^	2 (1–3)	1 (1–2)	0.02
History of prior termination of pregnancy	38 (40.4%)	40 (21.9%)	< 0.01
History of pre-gestational diabetes	1 (1.1%)	8 (4.4%)	0.28^c^
History of chronic hypertension	4 (4.3%)	9 (4.9%)	1.0^c^
Alcohol use in pregnancy	5 (5.3%)	6 (3.3%)	0.26
Tobacco use in pregnancy	28 (29.8%)	26 (14.2%)	< 0.01
Substance use in pregnancy	29 (30.9%)	16 (8.7%)	< 0.01
Prenatal depression	51 (54.3%)	78 (42.6%)	0.07
Sexually transmitted infection in pregnancy	17 (18.1%)	7 (3.8%)	< 0.01
Gestational age at presentation to prenatal care^b^	20 (14–25)	9 (7–11)	< 0.01

^a^all calculated using chi square test unless otherwise indicated

^b^value expressed as median (IQR) in place of n (%)

^c^Fisher’s exact test calculated in place of chi-square test

### Prenatal Care Utilization Score

The median score for contemplators was 17 (IQR 15–19) versus 18 (IQR 16–19) for non-contemplators (*P* < 0.01). A total of 62.8% of contemplators received a score of at least 16 points/21 possible points (~ 75% completion) on the guideline-based prenatal care utilization score, compared to 85.8% of non-contemplators (OR 0.28, 95% CI 0.16 – 0.50). Calculated retrospective power of this observed effect was 98% at alpha = 0.05. However this difference was no longer significant after adjusting for differences in maternal characteristics (aOR 1.0, 95% CI 0.40 – 2.56) (Table 2).

**Table 2:** Prenatal Care Utilization in Pregnant Women Who Consider but Do Not Have Abortions (Contemplators) Compared to Controls (Non-Contemplators)

Prenatal Care Utilization Score^a^	Contemplators n (%)	Non-Contemplators n (%)	*P*-value^b^	Odds ratio (95% CI)	Adjusted odds ratio (95% CI)^c^
*n* = 277	*n* = 94	*n* = 183			
Median score (IQR)	17 (15–19)	18 (16–19)	< 0.01	-	-
Score 75% (≥ 16 points)	59 (62.8%)	157 (85.8%)	< 0.01	0.3 (0.16 – 0.50)	1.0 (0.37 – 2.50)
Sub-analysis ^d^					
Median score (IQR)	11 (9–12)	11 (10–12)	0.11	-	-
Score 75% (≥ 11 points)	49 (52.1%)	110 (60.1%)	0.20	0.7 (0.44 – 1.19)	0.6 (0.24 – 1.29)

^a^Utilization was defined as adequate if the patient received 16 or more points/21 possible point (~ 75%)

^b^*P*-value calculated using chi-square test

^c^Adjusted for maternal characteristics of: race, age, insurance type, education, prior termination, tobacco use in pregnancy, substance use in pregnancy, depression in pregnancy, STI in pregnancy, and gestational age at presentation to prenatal care

^d^A sub-analysis was performed by removing two variables (“% of recommended prenatal visits received” and “first appointment by 12 weeks”) from the scoring system for a maximum score of 15

In the sub-analysis (removing “% of recommended prenatal visits received” and “first appointment by 12 weeks” from the scoring system), the median score for contemplators was 11 (IQR 9–12) and for non-contemplators was 11 (IQR 10–12), *P* = 0.11. There was no difference between groups in the frequency of achieving a ~ 75% completion rate/score of ≥11 (contemplators 52.1% versus non-contemplators 60.1%, *P* = 0.20).

### Individual measures of prenatal care utilization 

**Fig. 2:**
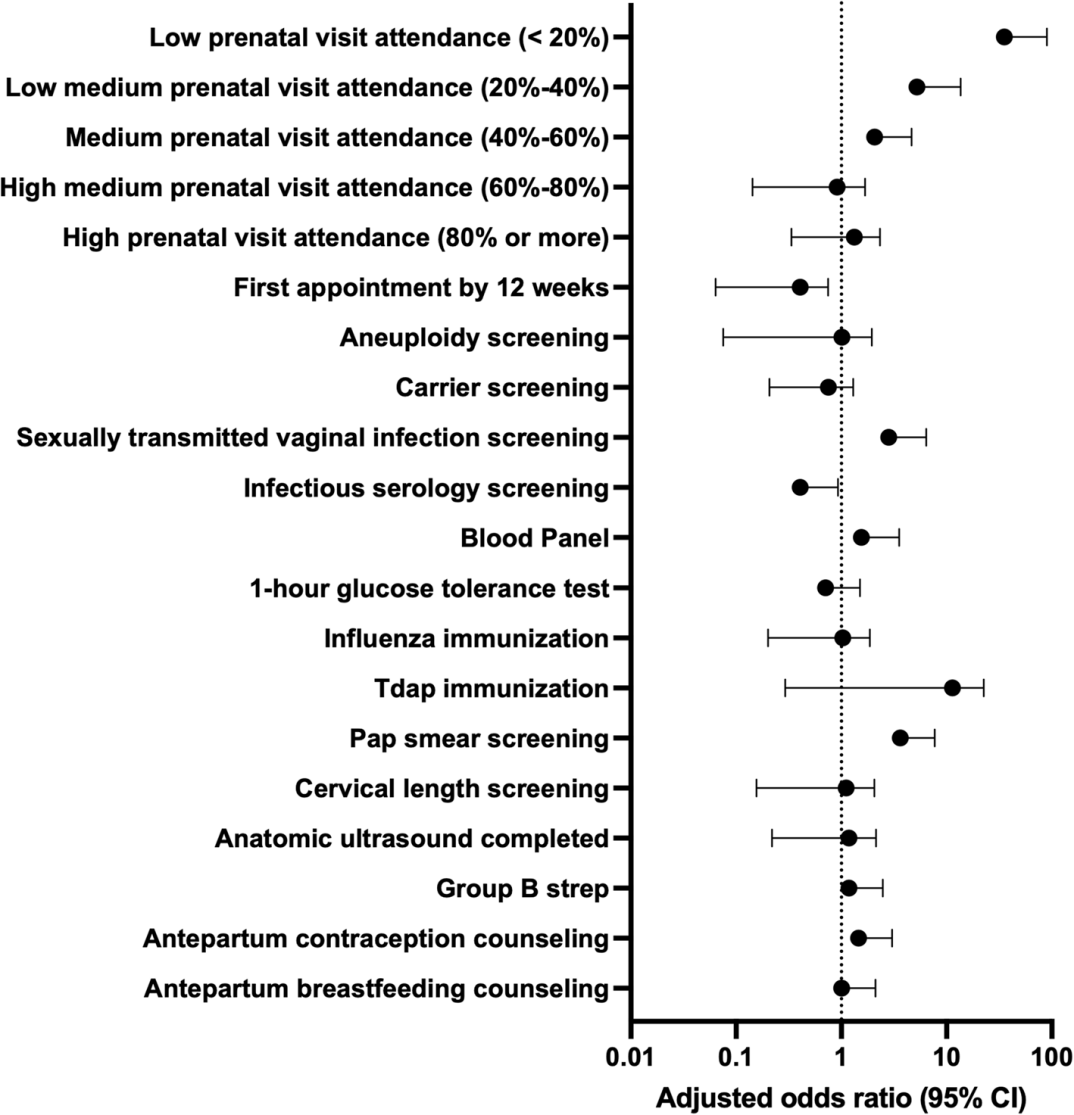
Forest Plot of Individual Measures of Prenatal Care Utilization in Pregnant Women Who Consider but Do Not Have Abortions (Contemplators) Compared to Controls (Non-Contemplators)

Contemplators were significantly less likely to present for prenatal care by 12 weeks gestation (contemplators 21.3% versus non-contemplators 82.5%, aOR 0.3, 95% CI 0.13 – 0.79). Non-contemplators were significantly less likely to receive Tdap immunization (contemplators 80.9% versus non-contemplators 60.1%, *p* < 0.01; aOR 7.8, 95% CI 2.53 – 23.8) (Table 3). After adjusting for confounding factors, there were no differences between groups in individual measures of prenatal care utilization (Fig. 2).

**Table 3:** Individual Measures of Prenatal Care Utilization in Pregnant Women Who Consider but Do Not Have Abortions (Contemplators) Compared to Controls (Non-Contemplators)

	Contemplators n (%)	Non-Contemplators n (%)	*P*-value^a^	Adjusted odds ratio^b^ (95% CI)
Total, *n* = 277	*n* = 94	*n* = 183		
Prenatal visit attendance				
High (80% or more)	60 (63.8%)	126 (68.9%)	0.40	1.1 (0.47 – 2.42)
High medium (60%-80%)	16 (17.0%)	41 (22.4%)	0.29	0.7 (0.27 – 1.76)
Medium (40%-60%)	9 (9.6%)	13 (7.1%)	0.47	1.0 (0.20 – 5.01)
Low medium (20%-40%)	3 (3.2%)	2 (1.1%)	0.21	0.8 (0.05 – 14.86)
Low (20% or lower)	6 (6.4%)	1 (0.5%)	< 0.01	7.5 (0.58 – 97.75)
First appointment by 12 weeks	20 (21.3%)	151 (82.5%)	< 0.01	0.3 (0.13 – 0.79)
Aneuploidy screening/genetic testing	59 (62.8%)	159 (84.2%)	< 0.01	0.7 (0.27 – 2.06)
Carrier screening	38 (40.4%)	96 (52.5%)	0.58	0.6 (0.30 – 1.36)
Sexually transmitted vaginal infection screening	91 (96.8%)	146 (79.8%)	< 0.01	1.3 (0.22 – 6.92)
Infectious serology screening	90 (4.3%)	175 (4.4%)	0.96	0.2 (0.02 -1.00)
Blood Panel	75 (79.8%)	179 (97.8%)	< 0.01	0.7 (0.13 – 3.83)
1-hour glucose tolerance test	73 (77.7%)	169 (92.3%)	< 0.01	0.4 (0.12 – 1.61)
Influenza immunization	57 (60.6%)	121 (66.1%)	0.37	0.8 (0.34 – 1.95)
Tdap immunization	76 (80.9%)	110 (60.1%)	< 0.01	7.8 (2.53 – 23.83)
Pap smear screening	87 (92.6%)	166 (90.7%)	0.61	2.1 (0.54 – 8.27)
Cervical length screening	70 (74.5%)	151 (82.5%)	0.11	0.8 (0.35 – 2.18)
Group B streptococcus screening	79 (84.0%)	164 (89.6%)	0.18	0.7 (0.20 – 2.65)
Anatomic ultrasound completed	62 (66.0%)	131 (71.6%)	0.62	0.9 (0.39 – 2.25)
Antepartum contraception counseling	73/88 (83.0%)	94/109 (86.2%)	0.52	0.9 (0.24 – 3.24)
Antepartum breastfeeding anticipatory guidance	75/88 (85.2%)	94/106 (88.7%)	0.48	0.6 (0.17 – 2.25)

^a^All *P*-values calculated using chi-square test

^b^Adjusted for maternal characteristics of: race, age, insurance type, education, prior termination, tobacco use in pregnancy, substance use in pregnancy, depression in pregnancy, STI in pregnancy, and gestational age at presentation to prenatal care

### Obstetric and neonatal outcome variables

**Fig. 3:**
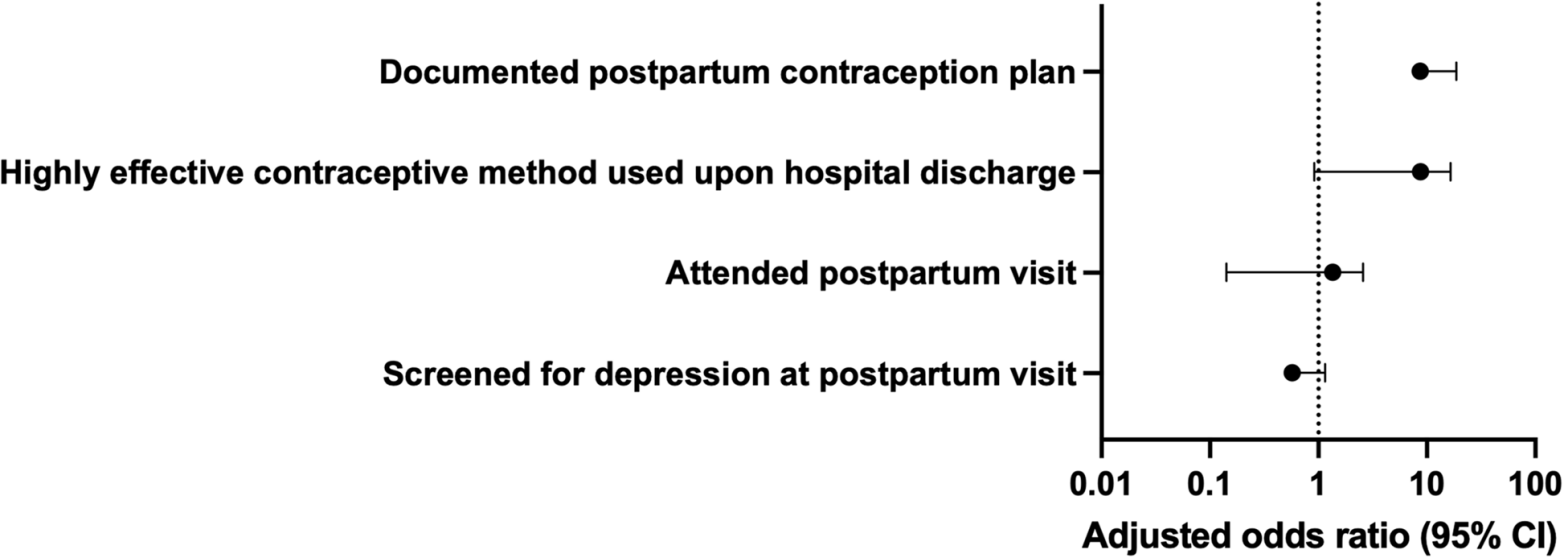
Forest Plot of Obstetric and Neonatal Morbidity in Pregnant Women Who Consider but Do Not Have Abortions (Contemplators) Compared to Controls (Non-Contemplators)

When adjusted for maternal demographic characteristics, there were no significant differences in odds of having obstetric or neonatal morbidity between contemplator and non-contemplator groups (Table 4, Fig. 3).

**Table 4:** Obstetric and Neonatal Morbidity in Pregnant Women Who Consider but Do Not Have Abortions (Contemplators) Compared to Controls (Non-Contemplators)

	Contemplators n (%)	Non-Contemplators n (%)	*P*-value^a^	Adjusted odds ratio^b^ (95% CI)
Total, *n* = 277	*n* = 94	*n* = 183		
Preterm prelabor rupture of membranes	10 (10.6)	11 (6.0)	0.17	1.3 (0.37 – 4.48)
Preterm delivery (< 37 weeks)	20 (21.3)	34 (18.6)	0.59	1.3 (0.55 – 3.23)
APGAR < 7 at 5 min	6 (6.6)	10 (5.5)	0.71	1.5 (0.33 – 6.81)
Low birth weight (< 2500 grams)	18 (19.4)	35 (19.1)	0.96	1.2 (0.49 – 2.83)
Neonatal intensive care unit admission	27 (28.7)	57 (31.1)	0.68	1.1 (0.48 – 2.30)
Placental abruption	8 (8.5)	4 (2.2)	0.14	4.7 (0.80 – 27.13)
Chorioamnionitis	4 (4.3)	6 (3.3)	0.68	1.3 (0.23 – 6.94)
Post-partum hemorrhage	7 (7.4)	9 (4.9)	0.40	1.2 (0.28 – 5.13)
Peri-partum blood transfusion	2 (2.1)	3 (1.6)	0.78	2.5 (0.17 – 36.17)

^a^All *P*-values calculated using chi-square test

^b^Adjusted for maternal characteristics of: race, age, insurance type, education, prior termination, tobacco use in pregnancy, substance use in pregnancy, depression in pregnancy, STI in pregnancy, and gestational age at presentation to prenatal care

### Postpartum care utilization

**Fig. 4:**
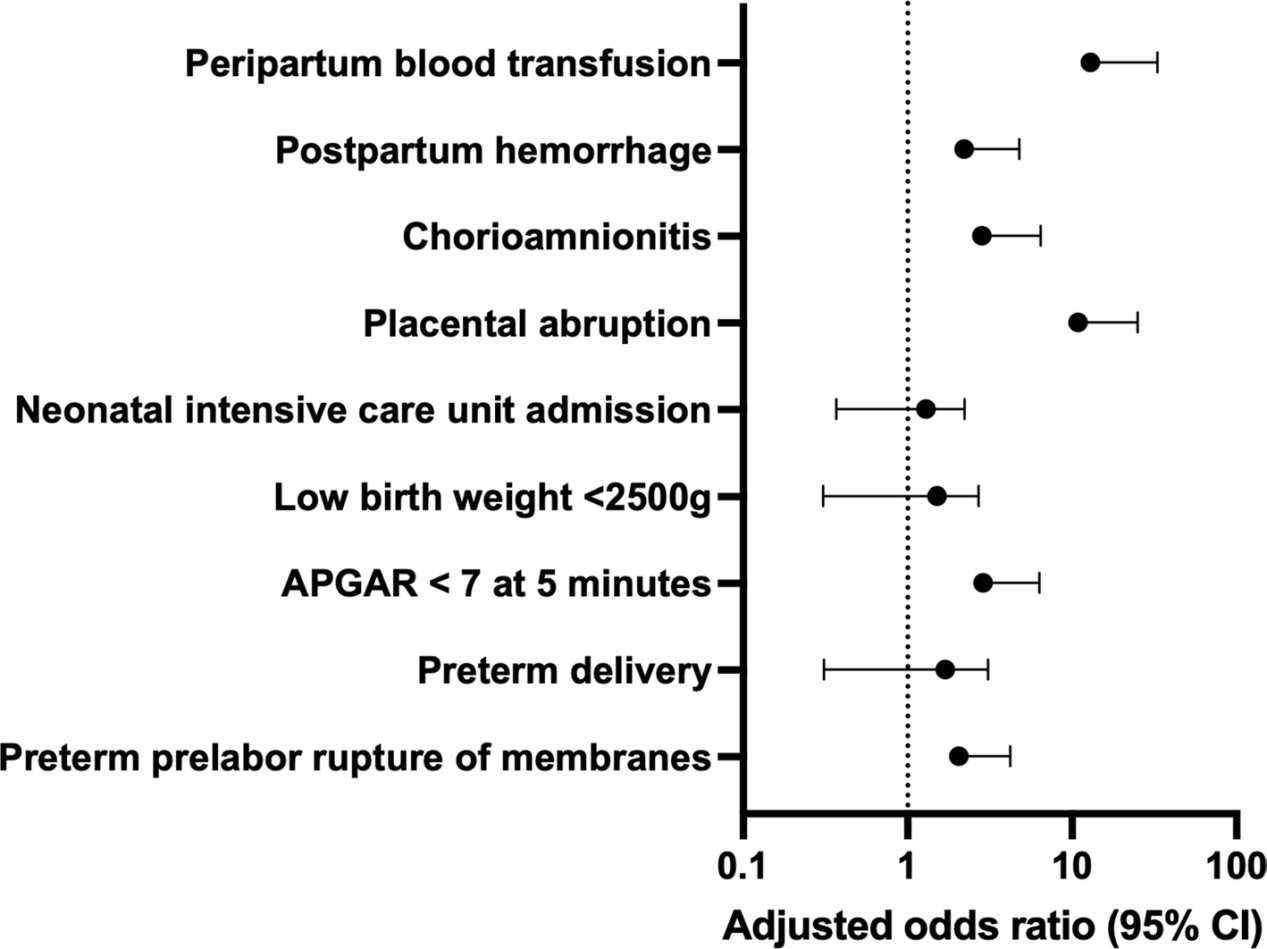
Forest Plot of Postpartum Care Utilization in Pregnant Women Who Consider but Do Not Have Abortions (Contemplators) Compared to Controls (Non-Contemplators)

Contemplators were significantly more likely to have immediate uptake of postpartum contraceptive plan prior to hospital discharge (contemplators 92.5% versus non-contemplators 79.4%; aOR 4.8, 95% CI 1.16 – 20.0) and to utilize a highly effective method upon discharge including permanent sterilization, long-acting reversible contraception, or administration of depot medroxyprogesterone acetate (contemplators 64.5% versus non-contemplators 79.4%; aOR 6.4, 95% C.I 2.34 – 17.4) (Table 5, Fig. 4).

**Table 5:** Postpartum Care Utilization in Pregnant Women Who Consider but Do Not Have Abortions (Contemplators) Compared to Controls (Non-Contemplators)

Characteristics	Contemplators n (%)	Non-Contemplators n (%)	*P*-value	Adjusted odds ratio^b^ (95% CI)
Documented postpartum contraceptive plan	86/93 (92.5%)	100/126 (79.4%)	< 0.01	4.8 (1.16 – 20.03)
Highly effective contraceptive method used upon hospital discharge^a^	60 (69.2%)	42 (42.0%)	< 0.01	6.4 (2.34 – 17.42)
Attended postpartum visit	50/85 (58.8%)	70/96 (72.9%)	< 0.01	1.0 (0.36 – 2.71)
Screened for depression at postpartum visit	13/61 (21.3%)	15/51 (29.4%)	0.32	0.4 (0.10 – 1.22)

^a^Includes long-acting reversible contraceptive placed or depo-provera administered prior to discharge, permanent sterilization, partner vasectomy

^b^Adjusted for maternal characteristics of: race, age, insurance type, education, prior termination, tobacco use in pregnancy, substance use in pregnancy, depression in pregnancy, STI in pregnancy, and gestational age at presentation to prenatal care

Non-contemplators were significantly more likely to attend a postpartum visit (contemplators 72.9% versus non-contemplators 58.8%, *P* < 0.01) though this did not remain significant after adjusting for differences in maternal demographic characteristics between groups (Table 5, Fig. 4).

## Discussion

A guideline-based prenatal care utilization scoring system developed for this study found that women contemplating pregnancy termination but ultimately continuing on to live birth initially appeared less likely to achieve ~ 75% completion of recommended prenatal care parameters than those who did not contemplate pregnancy termination, but this was not significant after adjustment. The strongest confounding factor in the adjusted regression was gestational age at presentation to prenatal care.

The prenatal care utilization scoring system did include variables related to timing of entry into prenatal care (“first prenatal care visit by 12 weeks” and “total number of prenatal care visits”). These time-based variables were included because authors were interested to know how prenatal care differed overall in contemplators as compared to the baseline population. However, it is well established in the literature that women with unplanned, mistimed, or unintended pregnancies present to prenatal care at a later gestational age and have fewer antenatal visits compared to those with planned pregnancies [10, 12, 23]. This was also demonstrated in the current study, where the adjusted odds of presenting to prenatal care by 12 weeks was 60% lower in contemplators compared to non-contemplators. Given this, a sub-analysis was performed removing these time-based variables from the prenatal care utilization scoring system to better understand how the quality of prenatal care delivered across different time frames might differ between groups. In the adjusted sub-analysis, there was no difference between groups in prenatal care utilization. These results demonstrate that although many women contemplating pregnancy termination present to prenatal care at a later gestational age, it appears they do “catch up” to the baseline population in achieving the objective components of adequate prenatal care. This is similar to what has been reported regarding prenatal care utilization among women with unintended pregnancies [27]. In addition, they do not experience increased rates of obstetric or neonatal morbidity, though the study is underpowered to demonstrate significant differences in these secondary outcomes.

Studies report that abortion turnaways have an increase in child-birth related morbidity and mortality as compared to those who are provided with abortions [28]. Although we did not detect any differences in the short-term, delivery-related maternal and neonatal morbidities between groups, we were unable to examine long-term outcomes in this study. Delayed maternal morbidity in parenting turnaways includes higher rates of socioeconomic hardship, worsening of self-reported health measures, lower educational attainment and lower rates of aspirational life plans [29–31]. Children born to parenting turnaways have higher rates of poor maternal bonding, lower childhood development scores, and are more likely to live below the poverty line [32, 33]. More research in this area is essential to better understand if there are other differences in prenatal care quality or delivery in women who consider but do not have an abortion for any reason, and how this may affect long-term outcomes in women, their children, and their families.

An interesting finding of the current study was that patients in the contemplator group had a significantly increased odds of using immediate post-partum contraception upon hospital discharge from their delivery admission and of using a highly-effective method of postpartum contraception. This persisted after adjusting for potential confounding variables and was present despite there being no difference between groups in rates of documented antepartum contraceptive counseling. Although the content of the antepartum contraceptive counseling is unknown in this case, these results suggest that the experiences of women who consider but do not complete an abortion is associated with their postpartum contraceptive uptake patterns. There were no differences between groups in adjusted rates of presentation to postpartum visit, which is consistent with other authors findings in this same population of women [23].

There are a number of limitations to the current study. The retrospective observational design introduces the possibility of bias related to inaccurate or incomplete record keeping. Certain prenatal care variables (in particular –immunization status for Tdap and Influenza) were not consistently documented by all providers in prenatal care records. An additional limitation is the possibility that some patients in the non-contemplator group did initially seek termination of pregnancy at an outside facility for which records were not available to the study team or may have contemplated TOP without pursuing an ultrasound related to that purpose. There are likely to be undetected differences in prenatal care utilization in subgroups of our study population based on individual reasons for not obtaining abortion (change in decision-making versus facing logistical, financial, or policy barriers to accessing abortion services), but these could not be determined using our retrospective design and would be better investigated by a future prospective survey and structured interview methodology.

## Conclusion

The current study recognizes that women considering TOP who ultimately continue the pregnancy to live birth often present later to prenatal care. Despite this, they still receive adequate guideline-based prenatal care as compared to women not considering pregnancy termination. Immediate uptake of postpartum contraceptive method and use of highly effective postpartum contraceptive methods was also higher in women considering but not completing abortion. Further research is necessary to better understand how prenatal care utilization and pregnancy outcomes are affected by the specific factors leading to women to consider but not complete abortions.

## Supplementary Information

**Additional file 1.** Flow diagram of subject selection.

## Data Availability

The datasets generated and/or analyzed during the current study are available in the Mendeley Data repository available at https://data.mendeley.com/datasets/r28gx9gzfr/1.

## Abbreviations

TOP: Termination of pregnancy
NYS-BC/SPDS: New York State Birth Certificate and Statewide Perinatal Data System
GED: General Educational Development Test
OR: Odds ratio
CI: Confidence interval
STI: Sexually transmitted infection
IQR: Interquartile range
TDap: Tetanus, Diphtheria, and Pertussis Immunization
