# Supplementary material for: Prenatal care utilization in pregnant women who consider but do not have abortions
Source: BMC Pregnancy Childbirth. 2022 Jan 21;22:53. doi: 10.1186/s12884-021-04343-x (PMC8780296; doi:10.1186/s12884-021-04343-x)
Supplement: Supplementary file 1 — Additional file 1. Flow diagram of subject selection. [file 12884_2021_4343_MOESM1_ESM.docx]

Identified by search terms as patient undergoing obstetrical ultrasound for termination of pregnancy

(n= 1676)

Excluded (n=335)

- Misclassified by search terms as ultrasound for termination of pregnancy (n=335)

Ineligible (n= 1247)

- Underwent termination of pregnancy (n=830)
- First trimester miscarriage or ectopic pregnancy (n=400)
- Incomplete records/lost to follow up (n=17)

Included in analysis

(n=94)

Assessed for inclusion

(n= 429)
